# Management of bipolar disorder in the intercontinental region: an international, multicenter, non-interventional, cross-sectional study in real-life conditions

**DOI:** 10.1038/srep25920

**Published:** 2016-05-16

**Authors:** Ludovic Samalin, Eduard Vieta, Tarek Ahmed Okasha, MM. Jalal Uddin, Seyed Ali Ahmadi Abhari, Fethi Nacef, Vyacheslav Mishyiev, Dovi Aizenberg, Yaël Ratner, Lydie Melas-Melt, Idir Sedeki, Pierre Michel Llorca

**Affiliations:** 1 CHU Clermont-Ferrand, EA7280, University of Auvergne, Clermont-Ferrand, France; 2 Bipolar Disorder Unit, Institute of Neuroscience, Hospital Clinic, University of Barcelona, IDIBAPS, CIBERSAM, Barcelona, Catalonia, Spain; 3 Institute of Psychiatry, Faculty of Medicine, Ain Shams University, Cairo, Egypt; 4 National Institute of Neurosciences and Hospital, Sher-e-Bangla Nagar, Dhaka, Bangladesh; 5 Roozbeh Hospital, South Kargar Street, Tehran, Iran; 6 Psychiatric Department, Razi Hospital, Manouba, Tunisia; 7 Kyiv City Clinical Psycho-neurological Hospital, Kiev, Ukraine; 8 Sackler Faculty of Medicine, Tel-Aviv University, Tel-Aviv, Israel; 9 Sha’ar Menashe Mental Health Center (MHC), Technion Institute of Technology, Haifa, Israel; 10 Biostatistician, Experis IT, Nanterre, France; 11 Sanofi, Avenue Raspail, Gentilly, France

## Abstract

Most of the existing data on real-life management of bipolar disorder are from studies conducted in western countries (mostly United States and Europe). This multinational, observational cohort study aimed to describe the management and clinical outcomes of bipolar patients in real-life conditions across various intercontinental countries (Bangladesh, Egypt, Iran, Israel, Tunisia, and Ukraine). Data on socio-demographic and disease characteristics, current symptomatology, and pharmacological treatment were collected. Comparisons between groups were performed using standard statistical tests. Overall, 1180 patients were included. The median time from initial diagnosis was 80 months. Major depressive disorder was the most common initial diagnosis. Mood stabilizers and antipsychotics were the most common drugs being prescribed at the time of the study. Antidepressants (mainly selective serotonin uptake inhibitors [SSRIs]) were administered to 36.1% of patients. Patients with bipolar I disorder received higher number of antipsychotics and anxiolytics than those with bipolar II disorder (p < 0.001). Presence of depressive symptoms was associated with an increase in antidepressant use (p < 0.001). Bipolar disorder real-life management practice, irrespective of region, shows a delay in diagnosis and an overuse of antidepressants. Clinical decision-making appears to be based on a multidimensional approach related to current symptomatology and type of bipolar disorder.

Bipolar disorder (BD) is a recurrent and chronic disease characterized by the occurrence of manic (or hypomanic), depressive, or mixed episodes. According to the World Health Organization, BD is one of the world’s ten most disabling conditions^1^. Several studies have shown that a considerable proportion of BD patients (30–60%) in clinical remission live with significant functional impairment^2^,^3^,^4^,^5^,^6^. In the general population, the estimated lifetime prevalence of BD is approximately 0.2–5% and increases to 6% for a broad range of bipolar spectrum disorders^7^,^8^,^9^,^10^. A first depressive episode, the presence of psychotic symptoms, and/or various comorbidities can dissimulate bipolar symptoms and may result in a delay in the diagnosis of BD. An estimated 35–45% of BD patients are misdiagnosed with unipolar depression^11^,^12^,^13^,^14^, and delays of up to 20 years from the onset of symptoms to the first mood stabiliser treatment have been reported^11^. Although the disease burden appears equivalent in both BD types^12^, the longitudinal course of patients with bipolar II disorder (age of onset, clinical course, predominant polarity, duration of episodes, and suicidality) differs from patients with bipolar I disorder^13^. Consequently, the risks of delayed diagnosis and misdiagnosis (as unipolar depressive disorder, personality disorders, or mild bipolar I disorder) are likely to be higher in bipolar II patients than in bipolar I patients.

Management of BD (type I or II) comprises complex treatment regimens to achieve the stabilisation of a mood episode and then the prevention of relapses or recurrences to allow for functional recovery. In recent decades, an increasing number of drugs, including lithium, anticonvulsants, and more recently second-generation antipsychotics, have been approved for the treatment of BD, presenting a new challenge for clinicians in choosing the most appropriate medication. Numerous guidelines have been developed by national agencies and professional organisations to guide clinicians to make their choice in the practice of appropriate evidence-based care^14^,^15^,^16^,^17^,^18^. However, many clinicians do not adhere to guidelines in routine practice, potentially due to negative attitudes toward guidelines (guidelines “are published by experts and not clinicians,” “are biased,” and “do not correspond to my patients” were some of the more telling responses from clinicians recently surveyed about their perceptions of the methods used in the evidence based-guidelines)^19^,^20^. Indeed, guidelines are often limited by the fact that they are typically based on the positive results of randomised controlled double-blind trials, which include BD patients only under restrictive criteria (e.g., monotherapy, exclusion of patients with medical or psychiatric comorbidities) and for a limited duration of assessment. Considering the gap between the highly selected, controlled evidence from research studies and the management of BD patients in real-life conditions, the applicability of guidelines in routine practice can be difficult to ascertain. Furthermore, the clinical management of BD patients can be affected by local customs, expert opinions, relationships with pharmaceutical industry, or politico-economic environments.

Most of the existing data on management of BD in real-life conditions are from studies conducted in western countries (particularly the United States and Europe)^21^,^22^,^23^,^24^,^25^,^26^,^27^,^28^,^29^,^30^,^31^,^32^,^33^. Few published trials have evaluated patients from other countries, and the data from these trials are very limited^34^. Therefore, the primary aim of the **MA**nagement of bi**P**olar disorder in **IN**tercontinental re**G**ion (MAPING) study was to provide information on the management of BD patients in conditions representative of routine clinical practice across different countries (Bangladesh, Egypt, Iran, Israel, Tunisia, and Ukraine) from the intercontinental region. Secondary objectives were to compare clinical outcomes and management of patients according to BD type and current bipolar symptoms.

## Methods

MAPING was an international, multicentre, non-interventional, cross-sectional study in adult BD patients conducted between September 2013 and May 2014. The study was performed in accordance with the Declaration of Helsinki and was carried out with the approved guidelines. All experiments were approved by Institutional Review Board or Institutional Ethics Committee from each participating country (Tehran university of medical science, Tehran, Iran; Razi hospital, La Manouba, Tunisia; Hadassah Hospital, Jerusalem, Israel; Kyiv City Clinical Psycho-neurological Hospital, Kiev, Ukraine; Sher-e-Bangla Hospital, Dhaka, Bangladesh; Ain Shams Hospital, Cairo, Egypt). All patients provided written informed consent to participate in the study.

### Selection of physicians

A feasibility study was conducted in potential study centres to confirm the capacity of potential sites to recruit the targeted study population. In order to ensure representativeness of the sample, 90 investigational sites (including psychiatric hospitals, general hospitals, and community office-based practices) were randomly selected from the pre-established list of study sites in Bangladesh, Egypt, Iran, Israel, Tunisia, and Ukraine. In the event that a physician declined to participate, s/he was replaced by the next physician on the randomised list.

### Patient selection

The study included patients aged ≥18 years fulfilling Diagnostic and Statistical Manual of Mental Disorders-Fourth Edition-Text Revision (DSM-IV-TR) diagnostic criteria for BD type I, type II, or not otherwise specified, and gave the informed consent. Exclusion criteria were concurrent enrolment in another study and presence of any acute physical condition. All consecutive eligible patients, at each site, were included in the study for a period of 8 months.

### Data collection

A site questionnaire collected the following information on the physicians: age, gender, specialty, years of practicing, main workplace (hospital, community, others), location, number of BD patients seen per month, knowledge and use of international and/or national guidelines (very frequently, from time to time, never).

Patients’ data were collected in a single visit by physician, who completed a case report form for each patient. Information collected included:

- Eligibility, date of visit and informed consent
- Patient characteristics: socio-demographic data (age, sex, area of residence, marital status, educational level, and medication health coverage), employment status, work disability, and physical comorbidities;
- Disease characteristics: type of BD, number of episodes and psychiatric hospitalizations during the last 12 months, predominant polarity of episodes (manic or depressive predominant polarity), family history of psychiatric history, age at initial diagnosis, initial diagnosis type, age at BD diagnosis, socio-familial and occupational functioning during the last 2 months (assessed by the Global Assessment of Functioning [GAF] scale^35^);
- Current bipolar symptoms listed in the DSM-IV-TR diagnostic criteria for BD: manic symptoms (feeling unusually “high” and optimistic or extremely irritable, grandiose beliefs about one’s abilities or powers, sleeping very little but feeling extremely energetic, talking so rapidly that others can’t keep up, racing thoughts, highly distractible or unable to concentrate, impaired judgment and impulsiveness, acting recklessly without thinking about the consequences, delusions, and hallucinations) and depressive symptoms (feeling hopeless or sad or empty, changes in appetite or weight, sleep disturbances, concentration and memory problems, feeling of worthlessness or guilt, thoughts of death or suicide, irritability, inability to experience pleasure, fatigue or loss of energy, and physical and mental sluggishness);
- Pharmacological treatment: treatment received at the time of initial diagnosis, antidepressant therapy at least once in life and if yes, the response to antidepressant (manic/hypomanic switches, resistance to treatment, mood lability, irritability, and unknown) and ongoing pharmacological treatment (mood stabilisers including lithium and anticonvulsants, first or second-generation antipsychotics, antidepressants, and electroconvulsive therapy).

### Statistical methods

The primary endpoint was the description of BD management across various countries, which included pharmacological treatment received or ongoing, psychiatric hospitalizations during the last 12 months and other healthcare resources use.

Secondary endpoints included the description of patients’ and diseases’ characteristics.

Comparisons were performed among:

- BD type I versus type II for the duration between initial identification of a pathological state and BD diagnosis (months), employment status, work disability, GAF score during the last 2 months, and ongoing pharmacological treatment (defined by class).
- Current bipolar symptoms and previous use of antidepressant (lifetime).

All analyses were conducted on the analysis population, by country and overall. The analysis population consisted of all patients enrolled in the study who met all inclusion/exclusion criteria.

Descriptive statistics of continuous variables were presented as the number of data analyzed, mean, standard deviation, median, quartiles and extreme values. Descriptive statistics of categorical variables were presented as the number and proportion of patients in each category. Missing data were not counted in the percentage.

Statistical comparisons between groups were performed using Chi-square test or Fisher’s exact test for categorical variables. Quantitative variables were compared using the Student’s t-test when normality is verified or the Wilcoxon Mann-Whitney test otherwise. All analyses were two-tailed, and significance was set at p < 0.05.

All comparisons performed and all p-value calculated were provided only for descriptive purpose.

Bonferroni correction was applied for multiple testing (adjusted p = 0.0025).

Statistical analysis was performed using SAS version 9.2 (SAS Institute, Cary, NC).

## Results

Overall, 89 participating physicians in hospital- and office-based settings screened 1447 patients diagnosed with BD. Of these patients, 1180 (81.5%) were included in the trial as the eligible population for the statistical analysis. The main reason for non-inclusion was patient refusal. Table 1 summarizes the number of sites and participating physicians and the number of screened, included, and eligible patients.

### Study sample characteristics

#### Physician characteristics

The mean age of participating physicians (77.5% men) was 50.6 ± 8.2 years with an average of 21.4 ± 8.3 years of practice. The median number of BD patients seen per month was 40. Most physicians (82.2%) used international guidelines very frequently (49.4%) or from time to time (48.3%).

#### Patient characteristics

Mean age was 37.9 ± 13.2 years, and 52.8% of patients were men (Table 2). Most patients were married and living in urban area. Overall, 79.8% of patients had a secondary or higher level of education, ranging from 61.3% in Bangladesh to 98.6% in Ukraine. For 52, 9% of patients, the medication health coverage was as out of pocket. Over half of the patients were unemployed, of whom, 47.0% of patients were unemployed because of a work disability.

Few patients (<17%) had past or existing physical comorbidities: the most frequent comorbidities were obesity, cardiovascular disease, diabetes, and thyroid disorder. Moreover, less than 6% of patients had a medical or surgical history of respiratory diseases, migraine, infectious diseases, or renal insufficiency.

#### Disease characteristics

Overall, 72.2% and 25.7% of patients were diagnosed with BD type I and type II, respectively, (Table 2). The median time from initial diagnosis was 80 months (range: 0–608). Most patients (64.4%) were diagnosed before the age of 30. Major depressive disorder and BD were the predominant initial diagnoses (36.4% and 20.2%, respectively).

Most patients (80.8%) had experienced between one and three episodes in the last 12 months. The predominant polarity of episodes was manic (65.9%).

One third of patients had a first-degree relative suffering from BD.

Global assessment of functioning during the last two months revealed moderate social, familial, and occupational dysfunction with a mean GAF score of 54.7 ± 19.7.

### Therapeutic management of BD

#### Therapies received at time of initial diagnosis

Overall, 67.2% of patients had received antipsychotic drugs at initial diagnosis, among them 51.1% received first-generation and 51.6% second-generation (Table 3). Moreover, 44.4% of patients had received antidepressants at initial diagnosis, mainly selective serotonin reuptake inhibitors (SSRIs; 67.2%). Mood stabilizers and anxiolytics were prescribed at initial diagnosis to an equivalent number of patients (29.7% and 30.0%, respectively).

Among the 63.8% patients who had received antidepressant at least once in lifetime, 46.1% experienced manic or hypomanic switches, 30.3% experienced irritability, and 29.6% experienced mood lability.

#### Ongoing pharmacological treatment

At the time of the study 85.7% of patients were being treated with mood stabilizers, mostly anticonvulsants (84.3%), and 83.4% of patients were receiving antipsychotics with a predominance of second-generation antipsychotics (79.6%), (Table 3). Anxiolytics were the third most frequently received therapy in 51.4% of patients. Antidepressants were being administered to 36.1% of patients (mainly SSRIs).

#### Psychiatric hospitalizations

46.0% of patients had at least one psychiatric hospitalization in the last 12 months, (Table 3).

### BD management according to type of BD and current symptomatology

#### BD type I versus BD type II

The average time from initial diagnosis to BD diagnosis was shorter for BD I patients (median, 0.8 years) than for BD II patients (median, 2.2 years), (Table 4). There was no observed difference in employment status and work disability based on the type of BD. However, BD I patients had a lower GAF score than BD II patients (53.8 ± 20.0 versus 56.9 ± 18.5, p = 0.017). BD I patients received more first-generation antipsychotics and anxiolytics than BD II patients (p < 0.001). Second-generation antipsychotics were used by more patients suffering from BD II than BD I (91.8% versus 75.9%). BD II patients received more antidepressants than BD I patients (p < 0.001). SSRIs remained the most common antidepressant for BD I (76.4%) and BD II (80.1%).

#### Current bipolar symptoms and treatment

Current symptoms of mania were associated with an increase in antipsychotic drug prescription (e.g., from 78.7% to 91.2% for “unrealistic or grandiose beliefs about one’s abilities or powers” (p < 0.001)). Patients who reported “racing thoughts: jumping quickly from one idea to the next” or “sleeping very little, but feeling extremely energetic,” used more mood stabilizers than others (90.0% versus 83.2%, p = 0.001 and 88.6% versus 82.6%, p = 0.003, respectively). Mania was also associated with decrease in antidepressant drug prescription.

Existing symptoms of bipolar depression were associated with a decrease in antipsychotic drug prescription (except for “feelings of worthlessness or guilt”, “thoughts of death or suicide”, and “irritability” (p < 0.01)) and an increase in antidepressant drug prescription (e.g., from 21.5% to 73.3% for “hopelessness, sadness or emptiness feelings” (p < 0.001)).

Most of these results (except decrease of antipsychotic prescription according to the presence of depressive symptoms) remained significant after Bonferroni correction (p < 0.0025).

## Discussion

This is the primary report from the MAPING study, which was designed to describe the clinical management of BD under real-life conditions across 6 countries (Bangladesh, Egypt, Iran, Israel, Tunisia, and Ukraine) from the intercontinental region.

Our findings show that, in the countries included:

- There is frequent initial misdiagnosis as major depressive disorder with resultant delay in the diagnosis of BD;
- Preferential use of anticonvulsants and second-generation antipsychotics in the treatment of BD with an overuse of antidepressants;
- Decision-making about treatment is based on BD type and existing symptoms.

In spite of some cultural aspects such as difference in medication health coverage, this is greatly similar to the clinical management of patients with BD in western countries^24^,^25^,^26^,^27^,^28^,^29^,^30^,^31^,^32^,^33^.

The challenges in diagnosing BD at the onset of disease arise from its complex and heterogeneous clinical presentation. As the first mood episode is frequently a depressive phase for BD patients, major depressive disorder was the predominant initial diagnosis in our study. The lifetime prevalence of major depressive disorder is approximately 4 times that of BD spectrum disorders, and the clinical presentation of a bipolar depression episode does not differ significantly from unipolar depression^36^. Consequently, there is often a delay of several years between initial diagnosis and BD diagnosis, associated with an initial over-prescription of antidepressants in 44.4% of patients. This issue is even more problematic in BD II patients, who are less quickly identified and consequently receive more antidepressants than BD I patients. The challenges of accurately identifying BD patients in clinical practice are similarly reported in western countries^36^,^37^,^38^. Therefore, the systematic screening of depressive patients with accurate assessment scales to identify hypomanic episodes, and the consideration of clinical characteristics associated with bipolar depression (family history of BD, earlier onset of illness, seasonality, mood reactivity, switching on antidepressants, history of suicide attempt) needed to be implemented in routine practice in all countries over the world^13^,^36^,^39^,^40^. In the future, an integrative approach with combinations of objective biomarkers could help in the differentiation between BD and unipolar depression^41^.

Overall, the most commonly prescribed medications were anticonvulsants, second-generation antipsychotics, and anxiolytics (almost always benzodiazepines). Over one third of patients were treated with antidepressants in spite of the increased risks of manic switches, irritability, mood lability, or inadequate response to treatment. Previous studies in western countries found the same trends of prescription in the treatment of BD. Psychotropic medications mainly involved antidepressants, anticonvulsants and second-generation antipsychotics in the United States^28^,^30^ and the European countries^25^.

Although most participating psychiatrists regularly used international guidelines to facilitate decision-making about treatment for BD management, they reported a preference for certain drugs depending on the type of BD or current bipolar symptoms. The presence of manic symptoms was associated with an increased use of antipsychotics and a decreased use of antidepressants. Conversely, depressive symptoms were associated with an increased use of antidepressants and a decreased use of antipsychotics. BD I patients received more first-generation antipsychotics and anxiolytics and BD II patients received more SSRIs, antidepressants, and second-generation antipsychotics. These findings are again in accordance with western studies. Several observational studies found highest antidepressant use in depression and higher second-generation antipsychotic prescription in mania^25^,^30^,^32^. In European countries, the BD II patients received more antidepressants^33^ and the BD I patients were treated with more antipsychotics in monotherapy or combination^29^.

Although most guidelines have focused on the use of mood stabilizers and not antidepressants, which are never recommended as monotherapy for acute or maintenance BD therapy, the use of antidepressants for the treatment of BD patients is still controversial^42^. As per certain international guidelines, insufficient levels of evidence justify that SSRIs in combination with mood stabilizers should not be recommended in bipolar depression, whereas other guidelines are more flexible regarding the use of antidepressants, particularly for BD II patients^43^. Inconclusive evidence is also available to consider antidepressant in combination with a prophylactic agent in the prevention of depressive episode^42^. All guidelines recommend avoiding antidepressant in patients with rapid cycling or history of mood switch during their use. If recent studies showed the possible interest of modern antidepressants in BD type II patients with lower risk of switching than BD type I, larger prospective trials are needed to confirm these findings^44^,^45^. The lack of expert consensus, paucity of evidence, and variability in guideline quality^46^ may explain the frequent use of antidepressants in our study but also in western countries.

Another relevant finding is the low rate of lithium use in comparison with anticonvulsants and second-generation antipsychotics, despite the fact that all international guidelines consider lithium as a first-line treatment in the acute manic phase and for prophylaxis^47^. As described in previous western studies^19^,^25^,^26^,^27^,^30^, the use of lithium seems to be declining due to clinicians’ apprehensions regarding the risk of side effects or intoxication, the long onset of action, or the practicability. The decline may also be the result of the recent development of second-generation antipsychotics for treatment of BD, and the lack of promotion of lithium, which is a generic and old medication.

Despite the large sample size, this study has several limitations. The cross-sectional design does not allow analysis of the causal relationship between the outcomes associated with management of bipolar patients and the different variables tested. The study did not involve all countries from the Middle East, Africa, South Asia, or Eurasia but selected some countries from these regions. The results cannot be considered as representative of routine clinical practice of regions that were not included in this study. Therefore, the analyses performed did not directly compare these intercontinental regions to each other. The high rates of type I BD and manic predominant polarity in this study, even if their prevalence varied broadly across studies, represent another limitation, which may be related to a selection bias.

Finally, the assessment of BD management was limited to patients who had access to the mental health care system, and BD patients without access to such care were not assessed.

In conclusion, the MAPING study provides relevant information on the management of BD patients in conditions representative of everyday clinical practice across 6 countries from the intercontinental region. An analysis of the findings indicates broad similarities in clinical management of BD patients included and those more widely studied in western countries. The real-life management of BD, irrespective of the country or region, shows a delay in diagnosis and is associated with a substantial use of antipsychotics, anticonvulsants, and SSRIs. Moreover, clinicians appear to base their decision making on a multidimensional approach related to the presence of manic or depressive symptoms. Taken together, management of patients with BD II presented some specific differences compared to management of those with BD I.

## Additional Information

**How to cite this article**: Samalin, L. *et al*. Management of bipolar disorder in the intercontinental region: an international, multicenter, non-interventional, cross-sectional study in real-life conditions. *Sci. Rep.* **6**, 25920; doi: 10.1038/srep25920 (2016).

## Figures and Tables

**Table 1:** Recruitment plan.

Region	Country	Sites	Participating physicians	Screened patients	Included patients*
Middle East	Israel	8	8	126	120
	Iran	20	20	244	200
Africa	Tunisia	12	12	204	185
	Egypt	20	20	301	300
South Asia	Bangladesh	15	15	388	225
Eurasia	Ukraine	15	14	184	150
**Total**		**90**	**89**	**1447**	**1180**

^*^All included patients were part of eligible population.

**Table 2:** Patient and disease characteristics by country.

N (%)		Bangladesh	Egypt	Iran	Israel	Tunisia	Ukraine	Total
		225 (19.1)	300 (25.4)	200 (16.9)	120 (10.2)	185 (15.7)	150 (12.7)	1180 (100)
Patient’ characteristics								
Age (years), mean (SD)		31.6 (10.2)	34.4 (11.2)	38.0 (13.1)	47.8 (15.4)	41.5 (12.1)	42.0 (13.5)	37.9 (13.2)
Male, n (%)		132 (58.7)	166 (55.3)	83 (41.5)	73 (60.8)	94 (50.8)	75 (50)	623 (52.8)
Living in urban area, n (%)		110 (48.9)	179 (59.7)	171 (85.5)	96 (80.0)	139 (75.1)	123 (82.0)	818 (69.3)
Married, n (%)		121 (53.8)	136 (45.3)	97 (48.5)	43 (35.8)	96 (51.9)	69 (46)	562 (47.6)
Educational level, n (%)	Illiterate	16 (7.1)	29 (9.7)	6 (3.0)	2 (1.7)	8 (4.3)	0 (0.0)	61 (5.2)
	Primary	71 (31.6)	34 (11.3)	30 (15.0)	9 (7.5)	31 (16.8)	2 (1.3)	177 (15.0)
	Secondary	99 (44.0)	104 (34.7)	88 (44.0)	69 (57.5)	78 (42.2)	68 (45.3)	506 (42.9)
	University	39 (17.3)	133 (44.3)	76 (38.0)	40 (33.3)	68 (36.8)	80 (53.3)	436 (36.9)
Employed, n (%)		92 (40.9)	161 (53.7)	94 (47.0)	50 (41.7)	93 (50.3)	60 (40.0)	550 (46.6)
Medication health coverage, n (%)	Total	9 (4.0)	29 (9.7)	39 (19.5)	111 (92.5)	109 (58.9)	10 (6.7)	307 (26.0)
	Partial	28 (12.4)	24 (8.0)	146 (73.0)	0 (0.0)	39 (21.1)	12 (8.0)	249 (21.1)
	None	188 (83.4)	247 (82.3)	15 (7.5)	9 (7.5)	37 (20.0)	128 (85.3)	624 (52.9)
Co-morbidities, n (%)	Cardio-vascular	11 (4.9)	12 (4.0)	18 (9.0)	13 (10.8)	6 (3.2)	17 (11.3)	77 (6.5)
	Obesity	28 (12.4)	30 (10.0)	32 (16.0)	14 (11.7)	8 (4.3)	7 (4.7)	119 (10.1)
	Diabetes	14 (6.2)	12 (4.0)	13 (6.5)	9 (7.5)	2 (1.1)	1 (0.7)	51 (4.3)
	Thyroid disorders	6 (2.7)	6 (2.0)	31 (15.5)	11 (9.2)	2 (1.1)	1 (0.7)	57 (4.8)
Disease characteristics								
Type BD I, n (%)		192 (85.3)	197 (65.7)	157 (78.5)	104 (86.7)	137 (74.1)	65 (43.3)	852 (72.2)
Rapid cycler, n (%)		14 (6.2)	10 (3.3)	12 (6.0)	9 (7.5)	7 (3.8)	1 (0.7)	53 (4.5)
Manic predominant polarity of episodes, n (%)		194 (86.2)	216 (72.0)	122 (61.0)	68 (56.7)	105 (56.8)	73 (48.7)	778 (65.9)
Family history of BD among first degree relatives, n (%)		49 (21.8)	95 (31.7)	82 (41.0)	37 (30.8)	83 (44.9)	18 (12.0)	364 (30.8)
Age (years) at initial diagnosis, mean (SD)		27.3 (8.2)	26.3 (9.3)	25.3 (9.9)	30.9 (13.7)	29.7 (11.2)	32.9 (10.4)	28.2 (10.5)
Initial diagnosis type, n (%)	Major Depressive	61 (27.1)	99 (33.0)	83 (41.5)	44 (36.7)	74 (40.0)	69 (46.0)	430 (36.4)
	Bipolar Disease	99 (44.0)	41 (13.7)	45 (22.5)	30 (25.0)	11 (5.9)	12 (8.0)	238 (20.2)
	Brief Psychotic Disorder	35 (15.6)	62 (20.7)	27 (13.5)	13 (10.8)	38 (20.5)	13 (8.7)	188 (15.9)
	Schizophrenia	11 (4.9)	22 (7.3)	9 (4.5)	7 (5.8)	18 (9.7)	8 (5.3)	75 (6.4)
	Other	19 (8.4)	76 (25.3)	36 (18.0)	26 (21.7)	44 (23.8)	48 (32)	249 (21.1)
Time (months) between initial and BD diagnosis, mean (SD)		17.8 (30.5)	32.6 (60.5)	50.6 (84.1)	73.7 (111.5)	57.4 (77.1)	49.7 (77.3)	43.1 (74.4)
Functioning (GAF score) within the last 2 months, mean (SD)		45.9 (16.7)	57.1 (18.2)	53.3 (22.3)	50.1 (18.2)	64.0 (19.2)	57.1 (18.7)	54.7 (19.7)

Abbreviations: BD – Bipolar Disorder; N – number of patients; SD – standard deviation.

**Table 3:** Main outcomes evaluating the management of BD.

Treatment received at the time of initial diagnosis		
Antidepressants, n (%)	524 (44.4)	
Antipsychotics, n (%)	793 (67.2)	
Mood stabilizers, n (%)	350 (29.7)	
Anxiolytics, n (%)	354 (30.0)	
Previous response to antidepressant		
Antidepressant received at least once lifetime, n (%)	753 (63.8)	
Manic/hypomanic switches	347 (46.1)	
Irritability	228 (30.3)	
Mood lability	223 (29.6)	
Resistance to treatment	148 (19.7)	
Unknown	122 (16.2)	
Ongoing pharmacological treatment		
Antidepressants, n (%)	426 (36.1)	
SSRIs	333 (78.2)	
SNRIs	92 (21.6)	
TCAs	98 (23.0)	
Antipsychotics, n (%)	984 (83.4)	
FGA	403 (41.0)	
SGA	783 (79.6)	
Mood stabilizers, n (%)	1011 (85.7)	
Lithium	395 (39.1)	
Anticonvulsants	852 (84.3)	
Electroconvulsive therapy, n (%)	187 (15.8)	
Anxiolytics, n (%)	606 (51.4)	
Benzodiazepines	588 (97.0)	
Other	43 (7.1)	
Psychiatric hospitalizations in last 12 months		
One, n (%)	408 (34.6)	
Multiple, n (%)	135 (11.4)	

Abbreviations: BD – bipolar disorder; FGA – first-generation antipsychotic; SGA – second-generation antipsychotic; SNRI – serotonin and norepinephrine reuptake inhibitors; SSRI – selective serotonin reuptake inhibitors; TCA – tricyclic antidepressants.

**Table 4:** Bipolar disorder type I and II comparisons.

	BD type I N = 852	BD type II N = 303	Total N = 1155	p-value	
Time between initial and BD diagnosis (months), mean (SD) [median]	37.3 (68.4) [9.0]	59.5 (87.2) [26.1]	43.1 (74.4) [12.0]	<0.001^*^	
Occupational status and productivity					
Employed, n (%)	387 (45.4)	151 (49.8)	538 (46.7)	0.095^**^	
Work disability#, n (%)	222 (47.7)	71 (46.7)	293 (47.5)	0.825^**^	
GAF score in the last 2 months, mean (SD)	53.8 (20.0)	56.9 (18.5)	54.6 (19.6)	0.017^*^	
Associated therapy received					
Antidepressants, n (%)	237 (27.8)	176 (58.1)	413 (35.8)	<0.001^**^	
SSRIs	181 (76.4)	141 (80.1)	322 (78.0)	0.364^**^	
SNRIs	43 (18.1)	45 (25.6)	88 (21.3)	0.068^**^	
TCAs	58 (24.5)	39 (22.2)	97 (23.5)	0.583^**^	
Antipsychotics, n (%)	747 (87.7)	219 (72.3)	966 (83.6)	<0.001^**^	
FGA	349 (46.7)	48 (21.9)	397 (41.1)	<0.001^**^	
SGA	567 (75.9)	201 (91.8)	768 (79.5)	<0.001^**^	
Mood stabilisers, n (%)	733 (86.0)	261 (81.6)	994 (86.1)	0.964^**^	
Lithium	295 (40.2)	93 (35.6)	388 (39.0)	0.189^**^	
Anticonvulsants	622 (84.9)	213 (81.6)	835 (84.0)	0.219^**^	
Electroconvulsive therapy, n (%)	138 (16.2)	46 (15.2)	184 (15.9)	0.678^**^	
Anxiolytics, n (%)	467 (54.8)	125 (41.3)	592 (51.3)	<0.001^**^	

Abbreviations: BD – Bipolar disorder; FGA – first-generation antipsychotic; GAF – Global Assessment of Functioning scale; N – number of patients; SD – Standard Deviation; SGA – second-generation antipsychotic; SNRI – serotonin and norepinephrine reuptake inhibitors; SSRI – selective serotonin reuptake inhibitors; TCA – tricyclic antidepressants.

*p-value, Wilcoxon-Mann-Whitney test (Two-sided).

**p-value, Chi-square test.

#Only for the unemployed patients.

## References

[b1] World Health Organization. *The Global Burden of Disease: 2004 Update.* (2008) Available at:http://www.who.int/healthinfo/global_burden_disease/2004_report_update/en/ (Accessed: 28th September 2015).

[b2] Samalin L. . Residual symptoms and functional performance in a large sample of euthymic bipolar patients in France (the OPTHYMUM study). J. Affect. Disord. 159, 94–102 (2014). 24679396 10.1016/j.jad.2014.02.023

[b3] Sanchez-Moreno J. . Functioning and disability in bipolar disorder: an extensive review. Psychother. Psychosom. 78, 285–297 (2009). 19602917 10.1159/000228249

[b4] Bauer M. S., Kirk G., Gavin C. & Williford W. O. Determinants of functional outcome and healthcare costs in bipolar disorder: a high-intensity follow-up study. J. Affect. Disord. 65, 231–241 (2001). 11511403 10.1016/s0165-0327(00)00247-0

[b5] Kleinman L. . Costs of bipolar disorder. Pharmaco-economics 21, 601–622 (2001). 10.2165/00019053-200321090-00001 12807364

[b6] Bryant-Comstock L., Stender M. & Devercelli G. Health care utilization and costs among privately insured patients with bipolar I disorder. Bipolar Disord. 4, 398–405 (2002). 12519100 10.1034/j.1399-5618.2002.01148.x

[b7] Kessler R. C. . Lifetime and 12-month prevalence of DSM-III-R psychiatric disorders in the United States. Results from the National Comorbidity Survey. Arch. Gen. Psychiatry 51, 8–19 (1994). 8279933 10.1001/archpsyc.1994.03950010008002

[b8] Weissman M. . Cross-national epidemiology of major depression and bipolar disorder. JAMA 276, 293–299 (1996). 8656541

[b9] Waraich P., Goldner E. M., Somers J. M. & Hsu L. Prevalence and incidence studies of mood disorders: a systematic review of the literature. Can J Psychiatry 49, 124–138 (2004). 15065747 10.1177/070674370404900208

[b10] Angst J. The emerging epidemiology of hypomania and bipolar II disorder. J. Affect. Disord. 50, 143–151 (1998). 9858074 10.1016/s0165-0327(98)00142-6

[b11] Hirschfeld R. M. Bipolar spectrum disorder: improving its recognition and diagnosis. J. Clin. Psychiatry 62, 5–9 (2001). 11469675

[b12] Merikangas K. R. & Lamers F. The ‘true’ prevalence of bipolar II disorder. Curr. Opin. Psychiatry 25, 19–23 (2012). 22156934 10.1097/YCO.0b013e32834de3de

[b13] Vieta E. & Suppes T. Bipolar II disorder: arguments for and against a distinct diagnostic entity. Bipolar Disord. 10, 163–178 (2008). 18199235 10.1111/j.1399-5618.2007.00561.x

[b14] Goodwin G. M..Evidence-based guidelines for treating bipolar bipolar disorder: revised third edition recommendations from the British Association for Psychopharmacology. J. Psycho-pharmacol. [cited 2016 Mar 15] doi:10.1177/0269881116636545. (2016). 10.1177/0269881116636545 PMC4922419 26979387

[b15] Grunze H. . The World Federation of Societies of Biological Psychiatry (WFSBP) guidelines for the biological treatment of bipolar disorders: update 2012 on the long-term treatment of bipolar disorder. World J. Biol. Psychiatry 14, 154–219 (2013). 23480132 10.3109/15622975.2013.770551

[b16] National Institute for Health and Clinical Excellence. Bipolar disorder: the assessment and management of bipolar disorder in adults, children and young people in primary and secondary care (2015) Available at: http://www.nice.org.uk/guidance/cg185/resources/guidance-bipolar-disorder-the-assessment-and-management-of-bipolar-disorder-in-adults-children-and-young-people-in-primary-and-secondary-care-pdf. (Accessed: 28th September 2015).

[b17] Yatham L. N. . Canadian Network for Mood and Anxiety Treatments (CANMAT) and International Society for Bipolar Disorders (ISBD) collaborative update of CANMAT guidelines for the management of patients with bipolar disorder: update 2013. Bipolar Disord. 15, 1–44 (2013). 23237061 10.1111/bdi.12025

[b18] Malhi G. S. . Clinical practice recommendations for bipolar disorder. Acta Psychiatr. Scand. Suppl. 439, 27–46 (2009). 19356155 10.1111/j.1600-0447.2009.01383.x

[b19] Samalin L., Guillaume S., Auclair C. & Llorca P. M. Adherence to guidelines by French psychiatrists in their real world of clinical practice. J. Nerv. Ment. Dis. 199, 239–243 (2011). 21451347 10.1097/NMD.0b013e3182125d4c

[b20] Perlis R. H. Use of treatment guidelines in clinical decision making in bipolar disorder: a pilot survey of clinicians. Curr. Med. Res. Opin. 23, 467–475 (2007). 17355728 10.1185/030079906X167444

[b21] Haro J. M. . Evidence for three distinct classes of ‘typical’, ‘psychotic’ and ‘dual’ mania: results from the EMBLEM study. Acta Psychiatr. Scand. 113, 112–120 (2006). 16423162 10.1111/j.1600-0447.2005.00692.x

[b22] Henry C. . A French network of bipolar expert centres: a model to close the gap between evidence-based medicine and routine practice. J. Affect. Disord. 131, 358–363 (2011). 21144593 10.1016/j.jad.2010.11.013

[b23] Sachs G. S. . Rationale, design and methods of the systematic treatment enhancement program for bipolar disorder (STEP-BD). Biol. Psychiatry 53, 1028–1042 (2003). 12788248 10.1016/s0006-3223(03)00165-3

[b24] Ghaemi S. N. . Pharmacological Treatment Patterns at Study Entry for the First 500 STEP-BD Participants. Psychiatr. Serv. 57, 660–665 (2006). 16675760 10.1176/ps.2006.57.5.660

[b25] Vieta E. . Clinical management and burden of bipolar disorder: results from a multinational longitudinal study (WAVE-bd). Int. J. Neuropsychopharmacol. 16, 1719–1732 (2013). 23663490 10.1017/S1461145713000278

[b26] Carlborg A., Ferntoft L., Thuresson M. & Bodegard J. Population study of disease burden, management, and treatment of bipolar disorder in Sweden: a retrospective observational registry study. Bipolar Disord. 17, 76–85 (2015). 25056132 10.1111/bdi.12234

[b27] Hayes J. . Prescribing trends in bipolar disorder: cohort study in the United Kingdom THIN primary care database 1995–2009. PLos One 6, e28725 (2011). 22163329 10.1371/journal.pone.0028725 PMC3233605

[b28] Baldessarini R., Henk H., Sklar A., Chang J. & Leahy L. Psychotropic medications for patients with bipolar disorder in the United States: polytherapy and adherence. Psychiatr. Serv. 59, 1175–1183 (2008). 18832504 10.1176/ps.2008.59.10.1175

[b29] Grande I. . Patterns of pharmacological maintenance treatment in a community mental health services bipolar disorder cohort study (SIN-DEPRES). Int. J. Neuro-psychopharmacol. 16, 513–523 (2013). 10.1017/S1461145712000405 22717099

[b30] Blanco C., Laje G., Olfson M., Marcus S. C. & Pincus H. A. Trends in the treatment of bipolar disorder by outpatient psychiatrists. Am. J. Psychiatry 159, 1005–1010 (2002). 12042190 10.1176/appi.ajp.159.6.1005

[b31] Walpoth-Niederwanger M. . Treatment patterns in inpatients with bipolar disorder at a psychiatric university hospital over a 9-year period: focus on mood stabilizers. Int. Clin. Psychopharmacol. 27, 256–266 (2012). 22842799 10.1097/YIC.0b013e328356ac92

[b32] Kessing L. V., Vradi E. & Andersen P. K. Nationwide and population-based prescription patterns in bipolar disorder. Bipolar Disord. 18, 174–182 (2016). 26890465 10.1111/bdi.12371

[b33] Karanti A., Kardell M., Lundberg U. & Landén M. Changes in mood stabilizer prescription patterns in bipolar disorder. J. Affect. Disord. 195, 50–56 (2016). 26859073 10.1016/j.jad.2016.01.043

[b34] Okasha T. A. . Longer-term treatment of patients with bipolar disorder: a 9-month observational study in Central and Eastern Europe, the Middle East and Africa. Curr. Med. Res. Opin. 25, 1889–1900 (2009). 19538106 10.1185/03007990903070270

[b35] Hall R. C. W. Global Assessment of Functioning: A Modified Scale. Psychosomatics 36, 267–275 (1995). 7638314 10.1016/S0033-3182(95)71666-8

[b36] Hirschfeld R. M. Differential diagnosis of bipolar disorder and major depressive disorder. J. Affect. Disord. 169, 12–16 (2014). 10.1016/S0165-0327(14)70004-7 25533909

[b37] Ghouse A. A., Sanches M., Zunta-Soares G., Swann A. C. & Soares J. C. Over diagnosis of bipolar disorder: a critical analysis of the literature. Scientific World Journal 2013, 297087 (2013). 24348150 10.1155/2013/297087 PMC3856145

[b38] Keck P. E., Kessler R. C. & Ross R. Clinical and economic effects of unrecognized or inadequately treated bipolar disorder. J. Psychiatr. Pract. 14, 31–38 (2008). 18677197 10.1097/01.pra.0000320124.91799.2a

[b39] Carvalho A. F. . Screening for bipolar spectrum disorders: A comprehensive meta-analysis of accuracy studies. J. Affect. Disord. 172C, 337–346 (2014). 25451435 10.1016/j.jad.2014.10.024

[b40] Mitchell A. J. Clinical utility of screening for clinical depression and bipolar disorder. Curr. Opin. Psychiatry 25, 24–31 (2012). 22139723 10.1097/YCO.0b013e32834de45b

[b41] Phillips M. L. & Kupfer D. J. Bipolar disorder diagnosis: challenges and future directions. The Lancet 381, 1663–1671 (2013). 10.1016/S0140-6736(13)60989-7 PMC5858935 23663952

[b42] Pacchiarotti I. . The International Society for Bipolar Disorders (ISBD) task force report on antidepressant use in bipolar disorders. Am. J. Psychiatry 170, 1249–1262 (2013). 24030475 10.1176/appi.ajp.2013.13020185 PMC4091043

[b43] Samalin L. . Methodological differences between pharmacological treatment guidelines for bipolar disorder: what to do for the clinicians? Compr. Psychiatry 54, 309–320 (2013). 23153855 10.1016/j.comppsych.2012.10.001

[b44] Amsterdam J. D. . Safety and effectiveness of continuation antidepressant versus mood stabilizer monotherapy for relapse-prevention of bipolar II depression: A randomized, double-blind, parallel-group, prospective study. J Affect. Disord. 185, 31–37 (2015). 26143402 10.1016/j.jad.2015.05.070 PMC4540653

[b45] Amsterdam J. D. & Shults J. Efficacy and safety of long-term fluoxetine versus lithium monotherapy of bipolar II disorder: a randomized, double-blind, placebo-substitution study. Am. J. Psychiatry 167, 792–800 (2010). 20360317 10.1176/appi.ajp.2009.09020284 PMC2896440

[b46] Castellani A., Girlanda F. & Barbui C. Rigor of development of clinical practice guidelines for the pharmacological treatment of bipolar disorder: systematic review. J. Affect. Disord. 174, 45–50 (2015). 25484176 10.1016/j.jad.2014.11.032

[b47] Nivoli A. M., Murru A. & Vieta E. Lithium: still a cornerstone in the long-term treatment in bipolar disorder? Neuro-psychobiology 62, 27–35 (2010). 10.1159/000314307 20453532

